# The impact of childbirth on gender wage gap: evidence from China

**DOI:** 10.3389/fsoc.2026.1887787

**Published:** 2026-10-16

**Authors:** Chong Guo, Xiaowang Pan, Yiqiu Wang, Meng Xie

**Affiliations:** 1 School of Business, Suzhou Vocational University, Suzhou, China; 2 School of Finance, Nanjing University of Agriculture, Nanjing, China; 3 School of Economics and Management, Henan Agricultural University, Zhengzhou, China

**Keywords:** childbirth, difference-in-differences, gender gap, motherhood penalty, wage

## Abstract

**Introduction:**

China’s evolving fertility policies have renewed academic attention to the impact of childbirth in the labor market. Whether the first and second birth transitions will lead to different changes in women’s and men’s wages is crucial for understanding the relationship between fertility behavior and gender pay gap.

**Methods:**

Based on the data of the four waves of China Household Finance Survey in 2013, 2015, 2017 and 2019, this study examines the impact of the first and second birth transitions on wages. The study uses a difference-in-differences framework and combines methods such as Callaway-Sant’Anna estimator, event study analysis, selection correction, and sensitivity tests.

**Results:**

The estimation results show a motherhood wage penalty and a fatherhood wage premium in the labor market, with larger wage changes for the second birth transition than for the first. Family support and digital finance appear to moderate these effects, and both help to reduce the motherhood wage penalty. Factors such as changes in working hours, employment interruption, and career progression may help explain the gender divergence in wages. Further analysis shows that the estimated wage effects are generally larger in absolute terms among workers outside the work unit system and those with non-establishment status. In the Oaxaca–Blinder decomposition, the observed individual characteristics can narrow the average gender wage gap, while the unexplained part widens it.

**Discussion:**

The findings indicate that childbirth-related wage differences are strongly gendered and become more pronounced around the second birth transition.

## 1 Introduction

The gender wage gap remains a persistent feature of labor markets. The International Labour Organization (2024), in its *Global Wage Report 2024-2025*, shows that women earn lower hourly wages than men in countries across all income groups. Some of the gap can be traced to differences in human capital and occupational allocation, but that does not fully explain the divergence between women’s and men’s wages through the family life cycle (Waldfogel, 1998; Blau and Kahn, 2017). Parenthood is central to this divergence. A substantial empirical literature shows a motherhood wage penalty, while the estimates for a fatherhood wage premium are less uniform and vary across countries, earnings groups, institutional environments, and empirical specifications (Budig and England, 2001; Gangl and Ziefle, 2009; Killewald, 2013; Cooke, 2014; Glauber, 2018; Mari, 2019). These asymmetric wage responses jointly contribute to widening the gender wage gap after childbirth. Labor market, organizational and household processes all contribute to this gender asymmetry. When childbirth happens, it can affect working time, employment continuity, career progression, and even the accumulation of work experience. At the same time, employer responses, along with gendered household specialization, may reinforce these changes (Mincer and Polachek, 1974; Fuegen et al., 2004; Correll et al., 2007; Miller, 2011; Goldin, 2014; Angelov et al., 2016; Kleven et al., 2019; Cortés and Pan, 2023). Such labor market adjustments tend to be larger and more persistent for women. Conversely, fathers’ changes in employment are less pronounced, and sometimes wages even increase (Killewald, 2013; Glauber, 2018; Mari, 2019).

Existing research on fertility and wages has largely examined the number of children, fertility timing, and birth spacing. Much of this literature focuses on women, investigating how specific childbearing patterns alter mothers’ wages and career trajectories (Budig and England, 2001; Anderson et al., 2003; Miller, 2011; Abendroth et al., 2014; Doren, 2019; Cukrowska-Torzewska and Matysiak, 2020; Hsu, 2021). There is some evidence that higher parity is associated with longer career interruptions, fewer working hours, and cumulative disadvantages in occupational status (Budig and England, 2001; Abendroth et al., 2014; Doren, 2019; Cukrowska-Torzewska and Matysiak, 2020). Some scholars have further paid attention to the heterogeneity of the wage impact across different birth orders, but the findings remain mixed. Cross country comparisons suggest that the motherhood wage penalty after the second child is larger than after the first child (Harkness and Waldfogel, 2003). However, evidence from Japan indicates short term earnings losses after the first and subsequent births, while longer term reductions in employment and wages are mainly tied to the first birth (Hsu, 2021).

China provides an informative setting for examining the impact of childbirth on wages. During the socialist era, the growth of women’s employment deepened women’s link to the labor market, but it never fully eliminated the gendered division of unpaid labor. Even when women were active in the labor market, in many cases, they still handled most of the domestic work and childcare (Zuo and Bian, 2001; Cook and Dong, 2011)^1^. At the same time, intergenerational childcare support is an important supplement to household care in China, which lightens childcare burdens for mothers, so that they can remain in paid work (Mu and Xie, 2016; Meng et al., 2023). Meanwhile, fertility restrictions were relaxed in stages, changing the institutional setting in which fertility decisions and family formation occur (Chen, 2023). On the urban labor market side, differences in work unit type and ownership segment employment into several institutional blocks, leading to systematic gaps in wages, employment stability, and workplace protections and benefits (Démurger et al., 2012; Jia and Dong, 2013). These institutional differences create distinct employment conditions for women and men, and also shape their wage trajectories after childbirth. Furthermore, the spread of digital finance and digital technologies has broadened women’s access to economic opportunities, and may have eased some of the frictions they face in juggling paid work and family responsibilities (Han et al., 2023; Qu and Xie, 2026).

Existing studies in China document motherhood wage penalties, gender gaps in employment and earnings after childbirth, and changes in the gender wage gap associated with the universal two-child policy (Jia and Dong, 2013; Ma, 2022; Zhou et al., 2022; Meng et al., 2023; Huang et al., 2024; Huang et al., 2025). Notably, both the size of the penalty and the extent of the gap differ across employer type, intergenerational childcare arrangements, and gender norms (Jia and Dong, 2013; Yu and Xie, 2018; Zhang M. et al., 2024). Much of the existing literature has concentrated on motherhood status, the number of children, specific employment or earnings outcomes, or the transition to parenthood. Yet evidence directly comparing women’s and men’s wage responses across successive birth transitions using the same data, outcomes, and empirical framework remains limited. Whether gender wage divergence varies by birth order therefore remains unresolved, as does whether any difference reflects changes in women’s wages, men’s wages, or both.

This paper empirically examines the wage effects of the first and second birth transitions for women and men in urban China. Using the 2013, 2015, 2017, and 2019 waves of the China Household Finance Survey, it estimates the motherhood wage penalty and fatherhood wage premium and evaluates their combined implications for the gender wage gap. It distinguishes the transition from no children to one child from the transition from one child to two children and estimates the corresponding wage effects separately by gender. Comparing the estimates for women and men allows us to determine whether the motherhood penalty and fatherhood premium differ between the two transitions and how these differences relate to gender wage divergence. Although China’s fertility policy changes form the context for this analysis, the focus is on individual childbirth transitions rather than policy effects.

This study makes three contributions relative to prior work. First, previous research on childbirth has generally viewed the transition to parenthood as a single event, with less attention to whether wage effects differ by birth order. This study develops an empirical strategy that distinguishes the first and second birth transitions and compares the estimated wage changes for women and men. Second, the analysis places women’s and men’s wage responses in the same empirical framework. This makes it possible to compare the motherhood wage penalty and the fatherhood wage premium directly and to assess what the two imply for gender wage divergence. Third, existing studies mainly examine the direct effects of family support, childcare, and digital development on women’s employment and income, but pay less attention to whether these factors alter wage outcomes after childbirth. This paper tests family support and digital finance as moderators of the wage effects of the first and second transitions. Digital finance is measured at both the individual and regional levels, bringing family resources and the digital environment into one framework.

The remainder of this paper is organized as follows. Section 1 reviews the existing literature and develops the research hypotheses. Section 2 describes the research design, while Sections 3 and 4 present the empirical results and further analyses. Finally, Section 5 concludes the paper.

## 2 Literature review and research hypotheses

### 2.1 Literature review

Early research on the wage effects of parenthood focused mainly on the motherhood wage penalty, with studies showing that mothers tend to earn less than otherwise comparable women without children, even after accounting for observed human capital and family characteristics. The size of this penalty differs across institutional and labor market settings, as well as by parity, education, and fertility timing (Waldfogel, 1998; Budig and England, 2001; Cukrowska-Torzewska and Matysiak, 2020; Doren, 2019; Yu and Kuo, 2024). Evidence on fertility timing is mixed. Some studies find that women who have their first child later lose less in hourly wages, while Austrian administrative data suggest that delaying motherhood may raise cumulative career earnings primarily by postponing the child penalty, rather than by reducing its relative magnitude or persistence (Bíró et al., 2026). Quasi-experimental studies exploiting plausibly exogenous fertility variation estimate substantial effects on women’s labor supply, earnings, and career trajectories following childbirth or an increase in family size (Angrist and Evans, 1998; Jacobsen et al., 1999; Agüero and Marks, 2008; Lundborg et al., 2017). For men, comparable estimates point to much smaller changes in labor supply (Angrist and Evans, 1998). Event study analyses also indicate that gender gaps in labor market outcomes widen after the first birth and often persist over time (Angelov et al., 2016; Kleven et al., 2019). Harmonized pseudo event studies covering 134 countries find widespread but heterogeneous employment penalties for women (Kleven et al., 2025).

Another line of research treats the pay consequences of parenthood as something that builds over a career. As households reorganize after marriage and childbirth, gender differences in earnings can unfold across the family life cycle (Juhn and McCue, 2017). Evidence from life cycle studies and linked data on employers and employees links these differences to employment interruptions, foregone earnings, skill depreciation, occupational sorting, employer changes, and wage growth within firms (Wilner, 2016; Adda et al., 2017; Yu and Hara, 2021). Even when mothers’ earnings begin to recover as children grow older, those earlier career interruptions can still slow career progress, and the gaps with fathers may persist or widen (Goldin et al., 2024). For fathers, the wage premium seems to depend on family arrangements, earnings position, labor market institutions, and wage trajectories prior to parenthood. Some studies suggest these advantages build slowly rather than appearing immediately after birth (Killewald, 2013; Glauber, 2018; Mari, 2019; Gowen, 2023).

It is less clear whether these patterns shift with birth order. Estimates by parity do not show a systematic rise in motherhood penalties with each additional child (Budig and England, 2001; Doren, 2019). Research from Japan and Norway documents declines in women’s employment or earnings in the short term following both first and second births (Hsu, 2021; Markussen and Strøm, 2022), but the longer term picture varies. Additional children are also associated with lasting career disadvantages among women, especially those with a college education (Cools et al., 2017). In Norway, in contrast, men’s labor supply and earnings rise after both transitions (Markussen and Strøm, 2022).

At the same time, research has examined public policy, the division of care work within households, and workplace arrangements that may reduce the motherhood wage penalty. Public childcare, parental leave, and other family policies can ease the tension between childrearing and employment, although their effects depend on policy design and the institutional environment (Budig et al., 2012; Olivetti and Petrongolo, 2017; Mari and Cutuli, 2021; Kleven et al., 2024). Job protection, the duration of parental leave, and how leave is shared between partners may work in different directions, with some effects offsetting others. Arrangements that support an earlier return to work and encourage partners to share leave may help limit mothers’ wage losses (Mari and Cutuli, 2021; Andersen, 2018; Patnaik, 2019). Family support can matter as well. Greater engagement of fathers in childcare, together with help from other family members, can ease mothers’ care burden and allow them to remain in the labor market (Posadas and Vidal-Fernandez, 2013; Arpino et al., 2014). Flexible working hours, telecommuting, and family-friendly measures may also reduce the costs to mothers of balancing work and childcare (Fuller and Hirsh, 2019; Bächmann et al., 2020). Digital technology provides a further source of support. Digital services could expand women’s economic opportunities and reduce the time and space barriers to combining paid work with family responsibilities. Digital finance can also improve access to financial services and help women remain economically active after childbirth (Guo et al., 2021; Han et al., 2023; Lu et al., 2023; Zhang C. et al., 2024).

Research in China expanded as employment became more market oriented. During the reform period, the motherhood wage penalty grew and was concentrated largely in the nonstate sector (Jia and Dong, 2013). Other studies linked additional children to more time in market work among fathers, while mothers’ hourly wage penalties varied with intergenerational coresidence (Mu and Xie, 2016; Yu and Xie, 2018). Motherhood penalties, fatherhood premiums, and wage differences among adults without children all contribute to the urban gender wage gap (Ma, 2022). More recent studies find wider gender differences in labor force participation after childbirth and persistent employment losses among mothers, with the size of these changes varying across historical periods and provinces (Zhou et al., 2022; Huang et al., 2025). Grandparental childcare is common in China and has been linked to stronger maternal labor market attachment (Du et al., 2019; Meng et al., 2023; Yu et al., 2023). Gender norms are partly shaped by Confucian family roles, which place greater responsibility for household work and childcare on women, and these norms are associated with larger child penalties (Ji et al., 2017; Zhang M. et al., 2024). Motherhood penalties tend to be smaller in occupations dominated by women, although these jobs generally offer lower pay, while fatherhood wage advantages remain evident across occupational types (Liu and Dai, 2026).

A large body of research has examined the wage consequences of childbirth for women and men and their implications for the gender wage gap. Recent work has considered the family and institutional conditions that may soften the motherhood wage penalty. However, evidence remains mixed on whether wage effects differ by birth order, and studies that directly compare women’s and men’s wage responses to first and second birth transitions in the Chinese context are especially scarce. Further research is therefore needed to distinguish between birth orders, compare women’s and men’s wage responses, and examine whether family support and digital finance are associated with differences in these wage effects.

### 2.2 Research hypotheses

Childbirth may affect women’s and men’s wages through gender-specific changes in household time allocation, employment continuity, and human capital accumulation (Cortés and Pan, 2023; Angelov et al., 2016; Kleven et al., 2019). Rising childcare demands create pressure to reallocate time from paid work to unpaid care, and women bear a disproportionate share of this adjustment since caregiving remains unevenly divided by gender. Women may therefore reduce their participation in paid work (Cortés and Pan, 2023; Andrew et al., 2024). Reductions in working hours and repeated or prolonged employment interruptions limit the accumulation of work experience, tenure, and firm-specific human capital, thereby weakening subsequent wage growth (Mincer and Polachek, 1974; Budig and England, 2001; Bertrand et al., 2010; Blundell et al., 2016; Adda et al., 2017; Andrew et al., 2024). Care responsibilities can also encourage moves to jobs that offer predictable hours and greater flexibility but fewer opportunities for wage growth (Miller, 2011; Wilner, 2016; Adda et al., 2017; Andrew et al., 2024). These career costs are likely to be greater in occupations where returns to working longer hours are nonlinear (Goldin, 2014).

Employer expectations and organizational practices can also reinforce these labor market adjustments (Correll et al., 2007; Halrynjo and Mangset, 2025; Choper, 2026). Under statistical discrimination, anticipated interruptions or limits on working time may enter employers’ assessments of workers’ future availability and expected productivity (Phelps, 1972; Correll et al., 2007). These expectations can shape hiring, job assignment, promotion, and wage growth, and they may also interact with how workers sort into firms and occupations (Francesconi, 2002; Miller, 2011; Wilner, 2016; Correll et al., 2007; Stojmenovska and England, 2021; Huang and Fan, 2025). Such effects are unlikely to be gender neutral. Norms that cast men as breadwinners tend to strengthen fathers’ attachment to paid work and encourage greater labor market investment, while caregiving norms make it more socially expected for mothers to reduce their time in paid work, with possible consequences for workplace decisions (Killewald, 2013; Baxter et al., 2015; Glauber, 2018; Olivetti and Petrongolo, 2017; Halrynjo and Mangset, 2025). Evidence from adoptive and same sex couples suggests that gendered child penalties do not arise from biological childbirth alone, but also reflect the institutional and normative organization of care (Andresen and Nix, 2022). In China, gender gaps in labor market outcomes after parenthood have been linked to gendered time allocation, care arrangements, and attitudes toward gender roles (Ma, 2022; Meng et al., 2023; Zhang M. et al., 2024; Huang et al., 2025; Li and Xiao, 2025). These patterns may contribute to lower wages for women and higher wages for men after childbirth, widening the gender wage gap.

Although the existing evidence is mixed, birth order may cut both ways. A second child may extend women’s employment interruptions, reduce time in paid work, and add to earlier losses in experience, tenure, and wage growth (Doren, 2019; Hsu, 2021; Markussen and Strøm, 2022). More children also mean greater financial obligations, which may strengthen fathers’ breadwinner role and labor market attachment (Killewald, 2013; Markussen and Strøm, 2022). Prior childcare experience, existing care arrangements, and economies of scale may soften the disruption created by another child (Hsu, 2021; Markussen and Strøm, 2022). These adaptive responses can ease some of the pressure from greater childcare demands, but the cumulative labor market effects of a second birth may remain (Adda et al., 2017; Cools et al., 2017). When caregiving responsibilities remain strongly gendered, the wage gap after a second birth may therefore be wider than after the first.

Based on the above analysis, this study proposes the following hypotheses:

*Hypothesis 1a*: Childbirth is associated with lower annual wages for women, generating a motherhood wage penalty.

*Hypothesis 1b*: Childbirth is associated with higher annual wages for men, generating a fatherhood wage premium.

*Hypothesis 1c*: The motherhood wage penalty and fatherhood wage premium jointly widen the gender wage gap.

*Hypothesis 2*: The motherhood wage penalty and fatherhood wage premium associated with the second birth transition are more pronounced than those associated with the first birth transition.

The labor market consequences of childbirth can vary with the institutional environment and with the economic and care resources available to families. Parental leave, public childcare, and other family-friendly policies may shape mothers’ employment and wages after childbirth (Olivetti and Petrongolo, 2017; Andersen, 2018; Mari and Cutuli, 2021). In addition, family economic resources can strengthen a household’s ability to cope with childcare costs and purchase care services, while care resources can reduce the extent to which new childcare demands crowd out mothers’ market work. Prior studies indicate that economic support and childcare support can improve women’s labor market outcomes by easing financial and time constraints, respectively (Bjorvatn et al., 2025). More affordable access to paid domestic services can increase women’s labor supply (Cortés and Pan, 2013). Care resources within the family also play a role; for example, grandparental childcare helps sustain women’s labor market participation (Yu et al., 2023). Furthermore, evidence from China and Germany shows that the availability of childcare services can improve mothers’ employment outcomes (Fang and Miao, 2024; Kronberg and Gerlach, 2026). Therefore, greater family economic and care resources may reduce the constraints that childbirth places on women’s labor market participation.

In addition to family and care resources, digital technology and digital finance may also ease the labor market constraints women face after childbirth by increasing time flexibility and improving access to financial resources. Internet technology can reduce the time and spatial constraints associated with some economic activities, and promote women’s labor market participation through remote work and time savings in household production (Dettling, 2017). Digital finance, for its part, can provide further support for women’s economic participation by lowering transaction and financing costs and improving access to financial services and economic opportunities. Prior research shows that fintech can ease financing constraints and promote women’s employment and entrepreneurship, which may help narrow the gender wage gap (Guo et al., 2021), while access to digital finance can strengthen women’s bargaining power within the household through greater participation in labor and financial markets (Han et al., 2023). Further studies find that broader digital economic development can promote women’s employment and narrow the gender wage gap (Lu et al., 2023; Zhang C. et al., 2024). Such development not only helps reduce the adverse labor market effects on women associated with marriage and childbearing (Xue et al., 2024), but also alleviates the motherhood penalty in China (Fang, 2026). Therefore, whether through individual use of digital financial services or a more developed digital finance and digital economy environment, women may be better positioned to maintain economic participation and reduce wage losses after childbirth.

Based on this, this study proposes hypothesis 3:

*Hypothesis 3*: Family support and digital finance moderate the wage effects of childbirth, and both can mitigate the motherhood wage penalty.

## 3 Research design

### 3.1 Data source and sample selection

This study uses the 2013, 2015, 2017, and 2019 waves of the China Household Finance Survey (CHFS), which is administered by the Survey and Research Center for China Household Finance at Southwestern University of Finance and Economics. The CHFS is based on a stratified, multistage probability sampling design and covers 29 provincial administrative units in mainland China, excluding Xinjiang and Tibet. The survey gathers detailed information on household composition, assets and liabilities, income and expenditure, insurance, social security, and individual employment characteristics. Its broad geographic coverage and detailed household information provide the data needed to examine household and labor market outcomes.

We restrict the sample to urban respondents with complete fertility information and estimate the wage effects of childbirth separately for women and men. The same marital status and retirement criteria were applied to both groups. Respondents who had never married or were cohabiting were excluded, as were those who had retired. Women aged 20–55 and men aged 22–60 were retained. These age ranges reflect the minimum legal marriage ages for women and men, together with the statutory retirement rules in force during the observation period^2^. China’s fertility restrictions were gradually relaxed during the study period, with the universal two-child policy implemented nationwide in 2016. The nationwide three-child policy was introduced only in 2021, after the 2013–2019 observation window. The analysis therefore focuses on the first and second birth transitions and excludes respondents with three or more children to align the analytical sample with the study’s focus on these transitions during the observation period.

Individual records were linked across survey waves using the available individual identifiers. When a respondent was observed in an earlier CHFS wave before the birth of the relevant child and again in a later wave after the birth, the earlier record provided directly observed pre-birth information. Respondents were not required to appear in all four waves; the resulting dataset is therefore an unbalanced individual panel rather than a balanced panel or a synthetic cohort. Among employed respondents, observations with missing wage information were excluded, whereas non-employed respondents were retained for the employment selection analysis. Observations with missing information on the core covariates were also excluded. To limit the influence of extreme values, continuous variables were winsorized at the 1st and 99th percentiles in the pooled sample. The master analytical sample contains 38,174 observations.

### 3.2 Empirical methods

#### 3.2.1 Baseline difference-in-differences specification

This study examines the effects of two childbirth transitions on conditional annual wages: the transition from no children to one child and the transition from one child to two children. Because the timing of childbirth differs across individuals, the treatment group and post-birth period are defined separately for each transition using the reported birth year of the relevant child. The baseline difference-in-differences specification compares changes in conditional annual wages before and after each transition between the relevant treatment and comparison groups, controlling for observed covariates and the specified fixed effects. We estimate separate models for women and men:

$$\begin{array}{l} \ln({\text{wage}}_{\mathrm{it}})=\alpha_{0}+\alpha_{1}{\text{treat}}_{\mathrm{it}}\times {\text{period}}_{\mathrm{it}}+\alpha_{2}X_{\mathrm{it}}\\ +{\text{city}}_{\mathrm{it}}+{\text{year}}_{t}+{\text{cohort}}_{i}+\varepsilon_{\mathrm{it}} \end{array}$$ (1)

In Equation 1, ${\text{wage}}_{\mathrm{it}}$ represents the annual wage of individual *i* in survey year *t*. The dependent variable is the natural logarithm of annual wages, calculated using $\text{wage}\_{\text{year}}_{\mathrm{it}}$ ^3^. The baseline wage regressions are estimated among employed respondents with valid wage information. The indicator for the period after the transition, ${\text{period}}_{\mathrm{it}}$ , is defined relative to the reported birth year of the relevant child and equals 1 in the year of the corresponding birth and in all subsequent survey years, and 0 in earlier survey years. ${\text{treat}}_{\mathrm{it}}$ identifies respondents who experience the relevant childbirth transition. We construct two schemes corresponding to the two birth transitions:

1. Scheme A examines the transition from no children to one child. Observations are retained only for periods in which respondents are either childless or have exactly one child. For respondents who subsequently have a second child, observations before the second birth are retained, whereas observations from the year of the second birth onward are excluded. Respondents who experience a first birth constitute the treatment group. Comparison observations consist of periods in which respondents have not yet experienced a first birth, together with observations of respondents who remain childless throughout the observation period.
2. Scheme B examines the transition from one child to two children. Observations are retained only for periods in which respondents have exactly one or two children. Respondents who experience a second birth constitute the treatment group. Comparison observations consist of periods in which respondents have not yet experienced a second birth, together with observations of respondents who remain at one child throughout the observation period.

In Equation 1, $\alpha_{1}$ is the key parameter of interest and measures the differential change in conditional annual wages between the treatment and comparison groups before and after the relevant childbirth transition. It serves as the baseline DID estimate of the wage effect of the corresponding childbirth transition. The control vector $X_{\mathrm{it}}$ includes individual characteristics, including age, education level, and health status; family characteristics, including the share of elderly household members and family assets; and regional macroeconomic variables, including industrial structure, the value-added share of the tertiary industry, general public budget expenditure, per capita GDP, and the proportion of tertiary sector employment. The terms ${\text{city}}_{\mathrm{it}}$ , ${\text{year}}_{t}$ , and ${\text{cohort}}_{i}$ denote the city, survey year, and cohort fixed effects^4^, respectively, while $\varepsilon_{\mathrm{it}}$ is the random disturbance term. Standard errors are clustered at the individual level to account for correlation across repeated observations of the same respondent. Detailed variable definitions are presented in Table 1.

**Table 1:** Variable definitions.

Variable name	Symbol	Definition
Annual wage	wage_year	Sum of after-tax monetary wages, bonuses, subsidies, and in-kind income during the survey year, unit: CNY/year
Age	age	Survey year - year of birth, unit: years
Square of age	age^2^	Square of age
Marital status	marriage	Married = 1; otherwise = 0
Education level		Reference category: bachelor’s degree or above
No formal schooling or primary education	edu_1	No formal schooling or primary school = 1; otherwise = 0
Junior high school	edu_2	Junior high school = 1; otherwise = 0
Senior high school or secondary vocational education	edu_3	Senior high school, secondary vocational school or technical school = 1; otherwise = 0
Junior college or higher vocational education	edu_4	Junior college or higher vocational education = 1; otherwise = 0
Health status		Reference category: poor or very poor health
Excellent	health_1	Excellent health = 1; otherwise = 0
Very good	health_2	Very good health = 1; otherwise = 0
Good	health_3	Good health = 1; otherwise = 0
Share of elderly household members	odr	The proportion of household members aged 65 or above, unit: %
Family assets	asset	Logarithm of total household assets^a^
Industrial structure	struc	The proportion of value added in the tertiary industry to that in the secondary industry
Share of value added in the tertiary industry	ti_add	The share of tertiary industry value added in regional GDP
Public budget expenditure	gov	Logarithm of general public budget expenditure
GDP per capita	gdp	Logarithm of GDP per capita
Share of employment in the tertiary industry	emp	Employment in the tertiary industry as a proportion of total employment

a In the CHFS database, total household assets include non-financial and financial assets. Non-financial assets include agricultural operating assets, industrial and commercial operating assets, land assets, real estate, vehicle assets, and other non-financial assets. Financial assets include social security account balances, cash, deposits, stocks, funds, bonds, derivatives, wealth management, foreign currency assets, gold, other financial assets, and lending.

To directly assess whether the estimated wage effect of childbirth differs by gender, we estimate a pooled specification with a gender interaction term:

$$\begin{array}{l} \ln({\text{wage}}_{\mathrm{it}})=\zeta_{0}+\zeta_{1}{\text{female}}_{i}+\zeta_{2}{\text{treat}}_{\mathrm{it}}+\zeta_{3}{\text{female}}_{i}\\ \times {\text{treat}}_{\mathrm{it}}+\zeta_{4}{\text{treat}}_{\mathrm{it}}\times {\text{period}}_{\mathrm{it}}+\zeta_{5}{\text{female}}_{i}\times {\text{treat}}_{\mathrm{it}}\\ \times {\text{period}}_{\mathrm{it}}+\zeta_{6}X_{\mathrm{it}}+{\text{city}}_{\mathrm{it}}+{\text{year}}_{t}+{\text{cohort}}_{i}+\varepsilon_{\mathrm{it}} \end{array}$$ (2)

In Equation 2, ${\text{female}}_{i}$ denotes whether the respondent is female. With men as the reference group, $\zeta_{4}$ represents the estimated wage effect for men, while $\zeta_{5}$ , the coefficient on the interaction with the female indicator, captures the corresponding gender contrast. The implied wage effect for women is given by $\zeta_{4}+\zeta_{5}$ . A statistically significant interaction coefficient $\zeta_{5}$ indicates that the estimated wage effect differs by gender. The specification is estimated separately for the first and second birth transitions.

#### 3.2.2 Group-time ATT estimation

Because individuals experience childbirth at different times, the baseline DID design features staggered treatment timing. When the effects of childbirth vary across treatment cohorts or survey periods, a single conventional DID estimate can pool comparisons across groups experiencing the transition at different times. To address this concern, we supplement the baseline DID estimates with the group-time average treatment effect on the treated (ATT) approach proposed by Callaway and Sant’Anna (2021). This approach calculates treatment effects for each cohort and survey year before combining them into an overall ATT. The group-time ATT is defined in Equation (3) as follows:

$${\mathrm{ATT}}_{\left(g,t\right)}=E[Y_{\mathrm{it}}(1)-Y_{\mathrm{it}}(0)|G_{i}=g]$$ (3)

Under Scheme A, *g* denotes the first survey year in which a respondent is observed at or after the first birth. Under Scheme B, the sample is restricted to respondents who have already had one child, and *g* denotes the first survey year in which a respondent is observed at or after the second birth. Consistent with the baseline DID design, respondents who have not yet experienced the relevant birth transition by year *t* serve as the comparison group. The group-time ATTs are obtained using a doubly robust procedure that combines outcome regression with inverse probability weighting. The resulting ATT (*g*, *t*) values are combined to produce the overall ATT and summarized by event time to trace dynamic wage effects around each childbirth transition. Although the linked CHFS data form an unbalanced individual panel, the CS-DID estimator is implemented using the repeated cross section form.

#### 3.2.3 Event time specification

To examine the dynamic wage effects at different points relative to childbirth, this study estimates an event study specification based on event time relative to the reported birth year, following Kleven (2022). Event time is defined as the difference between the survey year and the reported birth year of the relevant child. Under Scheme A, event time is measured relative to the birth year of the first child. Under Scheme B, it is measured relative to the birth year of the second child, with the sample restricted to respondents who had one child before the second birth transition. The corresponding event study specification is given by:

$$\begin{array}{l} \ln({\text{wage}}_{\mathrm{it}})=\beta_{0}+\sum_{\begin{array}{c} k=-3\\ k\neq -1 \end{array}}^{15}\beta_{k}[{\text{treat}}_{i}\times 1(\tau_{\mathrm{it}}=k)]\\ +\gamma X_{\mathrm{it}}+{\text{city}}_{\mathrm{it}}+{\text{year}}_{t}+{\text{cohort}}_{i}+\varepsilon_{\mathrm{it}} \end{array}$$ (4)

In Equation 4, $\tau_{\mathrm{it}}$ denotes the number of years between survey year *t* and the reported birth year of the relevant child, and 1 ( $\tau_{\mathrm{it}}=k$ ) is an indicator for event time *k*. The event window spans from three years before childbirth to 15 years after childbirth. The year immediately preceding childbirth, *k* = −1, is omitted as the reference period. For respondents in the comparison group who do not experience the relevant birth transition during the observation period, all treatment-by-event-time indicators are set to zero. The coefficients $\beta_{k}$ measure the differential change in conditional wages between the treatment and comparison groups at each event time, relative to the year immediately before childbirth. The pre-birth coefficients are used to examine whether differential wage changes are evident before childbirth, whereas the post-birth coefficients trace the evolution and persistence of the estimated wage effects following the transition. Because the analysis uses four CHFS survey waves and respondents are not observed continuously, the event time estimates reflect the available survey observations relative to the reported birth year rather than a continuously observed annual wage trajectory.

For comparison, we also aggregate the CS-DID group-time ATTs by event time. Given the four CHFS survey waves, these event time effects summarize changes observed at the available survey dates rather than continuous annual wage movements around childbirth. The baseline event study provides a finer event time profile based on reported birth years, whereas the CS-DID approach accommodates variation in treatment effects across childbirth cohorts and survey periods before aggregation.

### 3.3 Summary statistics

Table 2 reports descriptive statistics by gender and parity. For respondents who have valid wage information, childless women and men have similar mean log annual wages. The gender gap is significant among respondents with one child and is larger among those with two children. Annual earnings in the full analytical sample show much the same pattern. Women’s earnings trend downward as parity rises, while men with one or two children earn more on average than childless men. Employment patterns reveal a similar divide. Women with two children have the lowest employment rate, while employment among men increases with parity. The gender employment gap is therefore largest among respondents with two children. These unconditional differences across gender and parity groups do not identify the effect of an individual childbirth transition.

**Table 2:** Statistical descriptions of variables.

Variable	0 children^a^			1 child			2 children		
	Female	Male	*t*-test	Female	Male	*t*-test	Female	Male	*t*-test
Log wage_year	10.668	10.663	0.005	10.552	10.797	−0.245^***^	10.239	10.828	−0.591^***^
Annual earnings	39,093	43,834	−4741^***^	38,229	56,547	−18318^***^	19,499	60,047	−40548^***^
Employment rate	0.675	0.783	−0.107^***^	0.736	0.876	−0.140^***^	0.525	0.898	−0.373^***^
age	38.035	45.065	−7.030^***^	41.103	44.953	−3.850^***^	40.838	44.171	−3.333^***^
age^2^	1,558	2,175	−617^***^	1737	2082	−345^***^	1710	2003	−293^***^
marriage	0.740	0.784	−0.044^***^	0.960	0.981	−0.021^***^	0.985	0.987	−0.002
edu_1	0.069	0.053	0.016^***^	0.050	0.029	0.021^***^	0.125	0.078	0.047^***^
edu_2	0.175	0.212	−0.037^***^	0.213	0.192	0.021^***^	0.307	0.231	0.076^***^
edu_3	0.197	0.277	−0.0890^***^	0.260	0.286	−0.026^***^	0.25	0.277	−0.027^*^
edu_4	0.192	0.185	0.007	0.215	0.209	0.006	0.150	0.200	−0.050^***^
health_1	0.149	0.150	−0.001	0.136	0.141	−0.005	0.155	0.136	0.019^*^
health_2	0.380	0.350	0.030^***^	0.409	0.402	0.007	0.390	0.411	−0.021
health_3	0.272	0.302	−0.028^***^	0.327	0.327	0.000	0.315	0.312	0.003
odr	0.038	0.041	−0.003	0.038	0.037	0.001	0.037	0.039	−0.002
asset	13.262	13.283	−0.021	13.611	13.646	−0.035^*^	13.45	13.305	0.145^***^
struc	1.501	1.526	−0.025	1.445	1.480	−0.035^***^	1.339	1.370	−0.031
ti_add	0.517	0.525	−0.008^***^	0.516	0.521	−0.005^***^	0.503	0.506	−0.003
gov	8.513	8.528	−0.015	8.587	8.591	−0.004	8.571	8.535	0.036^*^
gdp	10.932	10.968	−0.036^***^	10.950	10.969	−0.019^***^	10.887	10.868	0.019
emp	0.457	0.466	−0.009^***^	0.453	0.459	−0.006^***^	0.438	0.437	0.001
Observations	4,720	6,783	11,503	10,729	11,656	22,385	2,250	2036	4,286

***, **, and * represent *p* < 0.01, *p* < 0.05, and *p* < 0.1, respectively. Data are from the 2013, 2015, 2017, and 2019 waves of the CHFS. All descriptive statistics are based on the master analytical sample, except for log annual wage, which is calculated among employed respondents with valid wage information. Annual earnings are coded as zero for non-employed respondents.

a Notably, 0 children means that the individual has not yet had any children by the time of observation, 1 child denotes that the individual has one child at the time of observation, and 2 children signifies that the individual has two children at the time of observation.

Parity groups also differ in observed characteristics. At each parity level, women are younger on average than men, and the groups vary in education, household resources, and regional economic conditions. The raw wage and employment gaps may therefore partly reflect dissimilarities in sample composition, which are controlled for in the regression analysis.

## 4 Empirical results

### 4.1 Baseline regression

Table 3 reports the estimated wage effects of the first and second birth transitions on log annual wages. Panel A presents the main results, with Columns (1)–(4) reporting the baseline DID estimates. For the first birth transition, annual wages decrease by approximately 10.95% for women and increase by 4.92% for men. The estimated wage change is larger after the second birth, reaching a 19.18% reduction for women and a 12.75% increase for men. These opposite wage responses fit the pattern of a motherhood wage penalty and a fatherhood wage premium, as expected under H1a and H1b. In the pooled specification, the gender interaction estimates are −0.167 for the first birth transition and −0.285 for the second, with both significant at the 1% level. Their negative signs indicate a less favorable wage effect for women, consistent with H1c.

**Table 3:** Baseline regression results.

Panel A. Baseline DID and CS-DID estimates								
Variable	DID				CS-DID			
	Scheme A (first birth transition)		Scheme B (second birth transition)		Scheme A (first birth transition)		Scheme B (second birth transition)	
	Female	Male	Female	Male	Female	Male	Female	Male
	(1)	(2)	(3)	(4)	(5)	(6)	(7)	(8)
Treat × Period	−0.116^***^ (−7.137)	0.048^***^ (3.489)	−0.213^***^ (−9.524)	0.120^***^ (7.789)				
ATT					−0.226^***^ (−2.75)	0.167^**^ (1.97)	−0.271^***^ (−3.99)	0.311^***^ (5.73)
Control variables	Y	Y	Y	Y	Y	Y	Y	Y
City FE	Y	Y	Y	Y	—	—	—	—
Year FE	Y	Y	Y	Y	—	—	—	—
Cohort FE	Y	Y	Y	Y	—	—	—	—
Observations	11,041	15,467	9,187	12,350	3,572	5,649	8,366	11,028
*R* ^2^	0.441	0.412	0.465	0.466	—	—	—	—
Gender difference (female minus male)	−0.167^***^		−0.285^***^		−0.393^***^		−0.582^***^	

Panel B. Cross transition differences		
Comparison	Female	Male
Second birth − first birth	−0.097^***^ (−3.39)	0.072^***^ (3.35)

***, **, and * represent *p* < 0.01, *p* < 0.05, and *p* < 0.1, respectively. In Panel A, robust *t*-statistics for the DID estimates and *z*-statistics for the CS-DID estimates are reported in parentheses. Standard errors for the DID estimates are clustered at the individual level. The bottom row reports the female minus male difference in the estimated childbirth effect. For the DID estimates, the gender contrast is estimated directly from the pooled gender interaction specification in Equation (1a) and corresponds to the coefficient on Female × Treat × Period. For the CS-DID estimates, the significance of the difference in the overall ATT between women and men is assessed using a two-sample *z*-test. In Panel B, the reported coefficients represent the difference between the second and first birth estimates, obtained from linear combinations of the estimated coefficients. *Z*-statistics are reported in parentheses. Percentage changes reported in the text are calculated from the log wage estimates as 100 [exp ( β ) − 1] and should be interpreted as approximate percentage changes in annual wages.

Columns (5)–(8) of Panel A report the CS-DID estimates. The estimates show a similar pattern when differences across childbirth cohorts and survey periods are taken into account. Annual wages fall by approximately 20.23% for women and rise by 18.18% for men after the first birth. After the second birth, the estimated changes are −23.74% for women and 36.48% for men. The corresponding gender contrasts are −0.393 and −0.582. The CS-DID estimates therefore retain the same gender asymmetry as the baseline results.

Panel B compares the wage effects of the first and second birth transitions using the baseline DID estimates. Relative to the first birth, the second birth estimate is 0.097 log points lower for women and 0.072 log points higher for men, with both differences significant at the 1% level. The larger motherhood wage penalty and fatherhood wage premium after the second birth support H2. The CS-DID estimates in Panel A show the same ordering, while the formal cross transition tests are reported in Panel B.

The motherhood wage penalty may partly reflect women’s greater childcare responsibilities. These responsibilities can reduce their labor supply, disrupt career progression, and slow human capital accumulation, while employer discrimination may add to these disadvantages. Fatherhood wage gains, on the other hand, may reflect breadwinner norms that strengthen men’s attachment to the labor market, along with employer attitudes that favor fathers.

### 4.2 Dynamic effects and parallel trends

The DID estimates in Table 3 rely on the conditional parallel trends assumption. In the absence of the relevant childbirth transition, the treatment and comparison groups should have followed similar conditional wage trends. We examine this assumption using an event study following Kleven (2022). Event time is measured relative to the reported birth year, with the comparison group defined separately for each scheme. The year immediately before childbirth, *k* = −1, is the reference period. The pre-birth coefficients are used to check for differences in wage trends before childbirth, and the post-birth coefficients show how the estimated wage effect changes relative to the reference period.

The baseline event study results for the first and second birth transitions are reported in Figure 1 for women and men separately. Across all four panels, the individual pre-birth coefficients are statistically indistinguishable from zero and show no systematic pattern. Joint tests also fail to reject the null hypothesis that the pre-birth coefficients are jointly zero. The corresponding *p* values are 0.987 and 0.965 for women and men in the first birth analysis and 0.413 and 0.546, respectively, in the second birth analysis. These results reveal no statistically detectable differential pre-trend over the observed pre-birth periods. Following childbirth, the coefficients generally become negative for women and positive for men relative to the omitted period, consistent with the gender asymmetry reported in Table 3. The post-birth point estimates are generally larger in absolute magnitude around the second birth transition, particularly for women.

**Figure 1:**
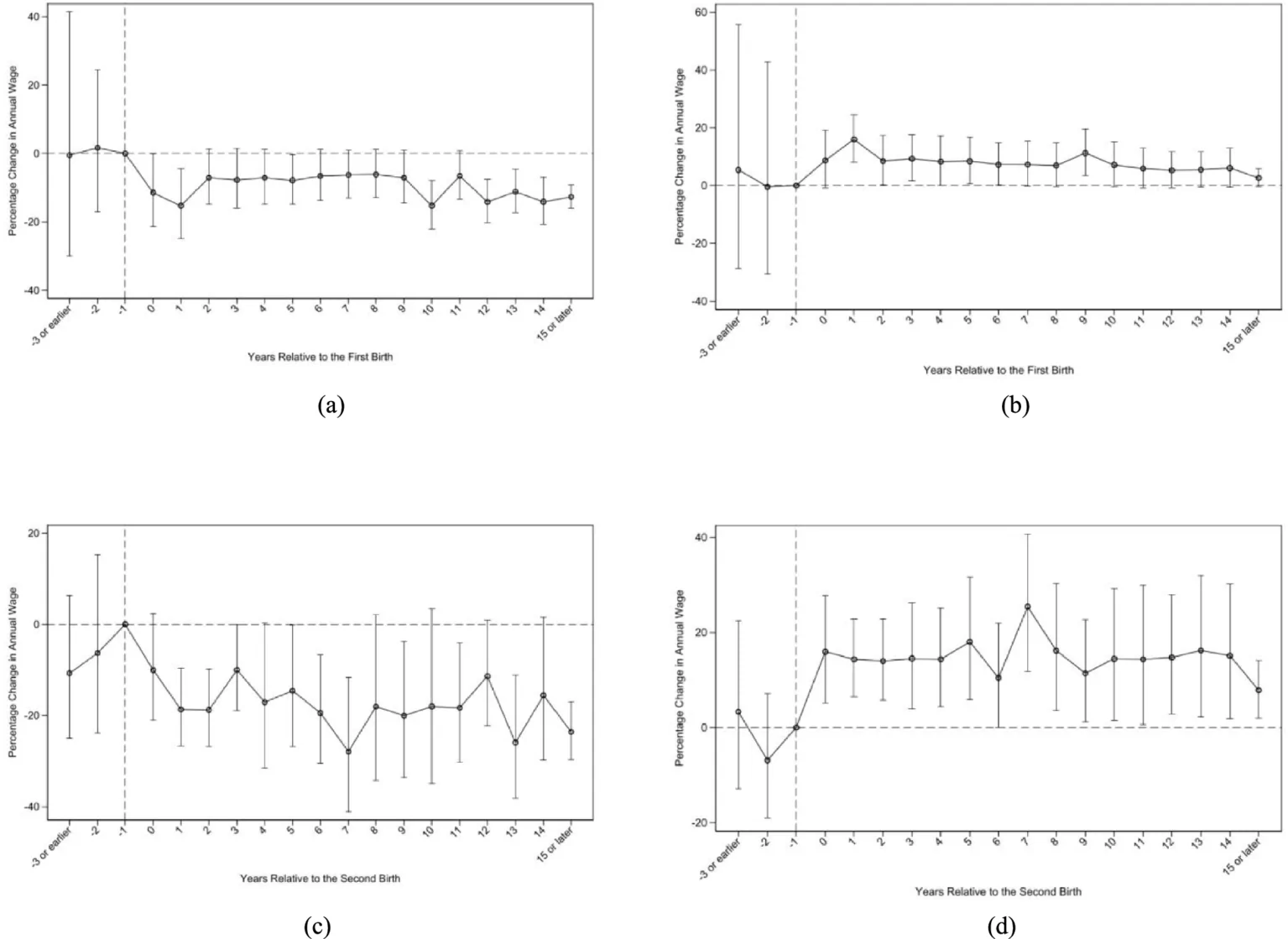
Baseline event study estimates. **(a)** Women: first birth transition. **(b)** Men: first birth transition. **(c)** Women: second birth transition. **(d)** Men: second birth transition.

Figure 2 reports the dynamic estimates obtained from the event time aggregation of the Callaway and Sant’Anna group-time ATT (*g*, *t*) values. The available pre-birth estimates are statistically indistinguishable from zero, whereas the post-birth estimates are generally negative for women and positive for men. This dynamic profile closely parallels the results in Table 3 and Figure 1, with generally larger point estimates around the second birth transition. Because the CS-DID analysis uses four CHFS survey waves, the number of observable pre-birth periods, and therefore the power of the pre-trend assessment, is limited. The pre-birth estimates nevertheless provide no evidence of differential trends over the observed pre-birth periods.

**Figure 2:**
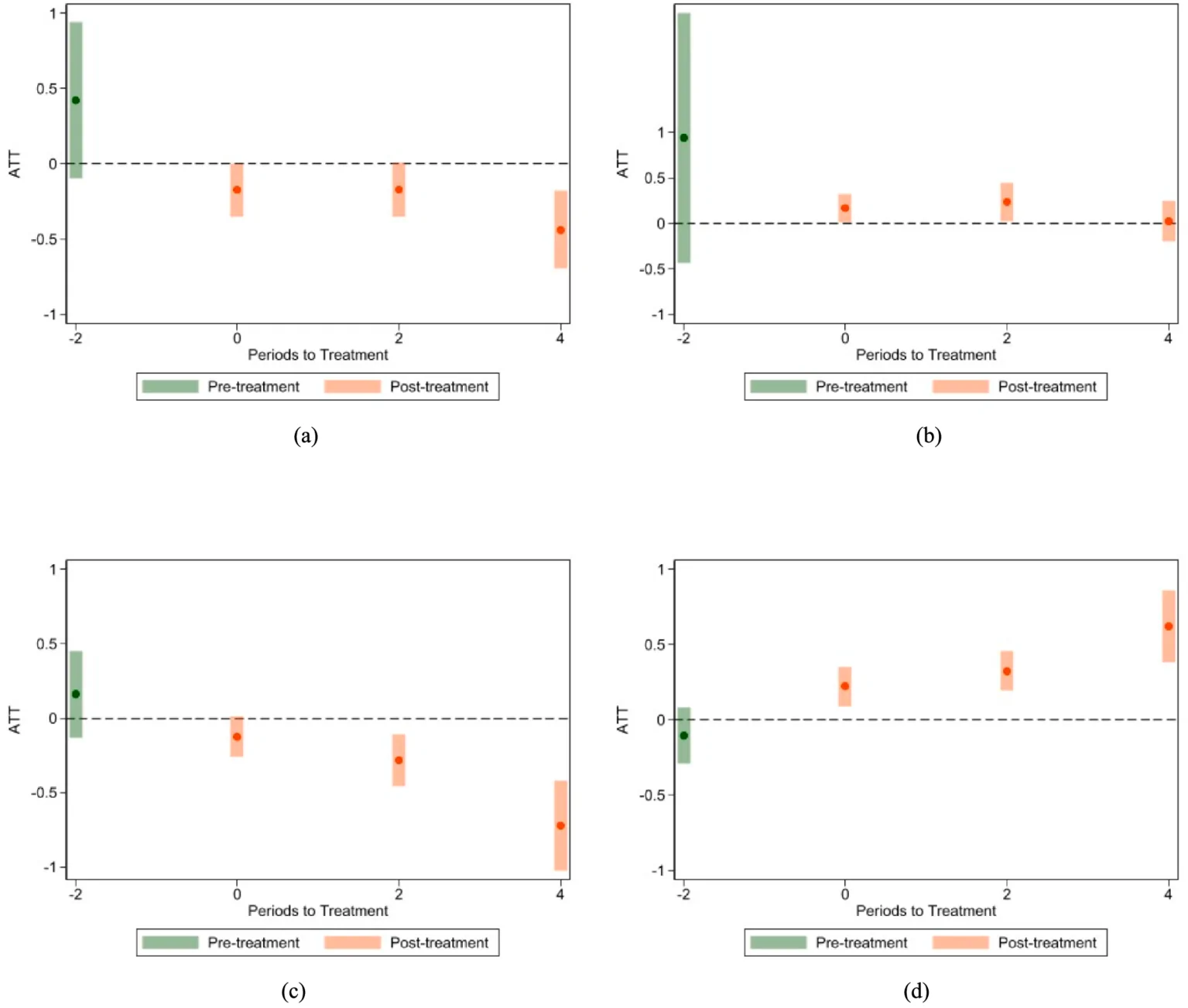
Callaway–Sant’Anna event time estimates. **(a)** Women: first birth transition. **(b)** Men: first birth transition. **(c)** Women: second birth transition. **(d)** Men: second birth transition.

### 4.3 Robustness checks

#### 4.3.1 Heckman selection correction

Annual wages are observed only for employed respondents. Accordingly, estimates based on the employed sample are conditional on employment and may be affected by nonrandom selection into the observed wage sample (Mulligan and Rubinstein, 2008). This concern applies to both women and men. Women may leave employment because of childbirth and childcare responsibilities. Men’s employment may also reflect unobserved factors, including ability, career preferences, health, and household resources. As a sensitivity analysis, we therefore estimate separate Heckman two-step selection models for women and men.

In the first stage probit model, employment status is regressed on the covariates used in the wage equation and two additional variables: intergenerational co-residence and other household income^5^. Intergenerational co-residence refers to living with the respondent’s parents or parents-in-law. It can facilitate employment participation through the availability of childcare and household support, whereas other household income may influence the financial need to engage in paid work. These variables are excluded from the wage equation under the maintained exclusion restriction that, conditional on observed individual, household, and regional characteristics, they affect annual wages only through employment selection. In the second stage, log annual wages are estimated among employed respondents, with the inverse Mills ratio derived from the first stage included to account for selection. Table 4 reports the results of the selection and wage equations, together with joint significance tests for the two exclusion variables conducted separately by gender.

**Table 4:** Heckman selection correction results.

Variable	Female		Male	
	Selection equation	Wage equation	Selection equation	Wage equation
	(1)	(2)	(3)	(4)
One child^a^	−0.054^*^ (−1.72)	−0.104^***^ (−6.540)	0.070^**^ (2.26)	0.036^***^ (2.725)
Two children^b^	−0.554^***^ (−12.98)	−0.265^***^ (−8.675)	0.393^***^ (6.96)	0.151^***^ (7.795)
Intergenerational co-residence	−0.079^*^ (−1.71)		−0.219^***^ (−4.17)	
Other household income	−0.029^***^ (−7.04)		0.022^***^ (5.18)	
IMR		−0.208^***^ (−2.605)		−0.152^**^ (−2.219)
Control variables	Y	Y	Y	Y
City FE	Y	Y	Y	Y
Year FE	Y	Y	Y	Y
Cohort FE	Y	Y	Y	Y
Observations	17,602	12,210	20,087	16,962
*R* ^2^	0.224	0.453	0.247	0.429
Joint test of exclusion variables, *χ*^2^ (2)	50.94, *p* < 0.001		40.18, *p* < 0.001	

***, **, and * represent *p* < 0.01, *p* < 0.05, and *p* < 0.1, respectively. Robust *t*-statistics based on standard errors clustered at the individual level are reported in parentheses. The reference category for childbirth status is respondents with no children.

a The one child indicator equals 1 if the respondent has one child at the time of observation and 0 otherwise.

b The two children indicator equals 1 if the respondent has two children at the time of observation and 0 otherwise.

The first stage results show that intergenerational co-residence and other household income significantly predict employment participation. For both women and men, the exclusion variables are jointly significant in the selection equation. The inverse Mills ratio enters the wage equation with a negative and significant coefficient in both the female and male wage samples, which points to nonrandom selection into the observed wage sample under the Heckman model. After incorporating the selection correction, the wage coefficients retain the direction and ordering of the baseline results. Relative to childless respondents, women with one or two children have lower estimated log annual wages, whereas the corresponding coefficients for men are positive. For both women and men, the coefficients are larger in magnitude among respondents with two children than among those with one child, consistent with the ordering of the baseline estimates. This analysis compares wage levels across parity groups rather than estimating changes in individual wages before and after childbirth. The persistence of the gender and parity pattern after the selection correction suggests that accounting for employment selection does not materially alter the baseline pattern. The Heckman estimates thus provide supplementary evidence that the qualitative pattern of lower wages among women with children and higher wages among men with children remains evident after accounting for selection into employment.

#### 4.3.2 Alternative wage measure

To assess whether the baseline results depend on the choice of wage measure, we re-estimated the models using the logarithm of hourly wages instead of the logarithm of annual wages. Annual wages reflect both the wage rate and the amount of paid work performed over the year, whereas hourly wages capture remuneration per unit of reported working time. The hourly wage specification therefore allows us to examine whether the estimated gender asymmetry is also present in wage rates rather than reflecting solely differences in total working time. Columns (1)–(4) of Table 5 report the results. The estimated signs remain unchanged from the baseline results.

**Table 5:** Additional robustness checks.

Variable	Alternative wage measure				Controlling for fertility policy differences			
	Scheme A (first birth transition)		Scheme B (second birth transition)		Scheme A (first birth transition)		Scheme B (second birth transition)	
	Female	Male	Female	Male	Female	Male	Female	Male
	(1)	(2)	(3)	(4)	(5)	(6)	(7)	(8)
Treat × Period	−0.089^***^ (−5.110)	0.065^***^ (4.455)	−0.152^***^ (−6.862)	0.107^***^ (6.022)	−0.116^***^ (−7.093)	0.047^***^ (3.437)	−0.213^***^ (−9.514)	0.121^***^ (7.799)
Policy1					Y	Y	Y	Y
Policy2					Y	Y	Y	Y
Control variables	Y	Y	Y	Y	Y	Y	Y	Y
City FE	Y	Y	Y	Y	Y	Y	Y	Y
Year FE	Y	Y	Y	Y	Y	Y	Y	Y
Cohort FE	Y	Y	Y	Y	Y	Y	Y	Y
Observations	11,041	15,467	9,187	12,350	11,041	15,467	9,187	12,350
*R* ^2^	0.437	0.393	0.457	0.432	0.441	0.412	0.465	0.466
Gender difference (female minus male)	−0.154^***^		−0.259^***^		−0.163^***^		−0.334^***^	

***, **, and * represent *p* < 0.01, *p* < 0.05, and *p* < 0.1, respectively. Robust t-statistics based on standard errors clustered at the individual level are reported in parentheses. The bottom row reports the difference in the estimated coefficient of the core explanatory variable between the male and female samples. The significance of this difference is assessed using empirical *p*-values obtained from 1,000 bootstrap replications.

#### 4.3.3 Controlling for the potential influence of different fertility policies

The initially stringent one-child policy was gradually eased to permit couples meeting specified conditions to have a second child in various provinces^6^. This was followed by the selective two-child policy and the universal two-child policy. Because the timing and implementation of these changes differed across provinces and survey years, the regressions account for these differences in fertility policy implementation. Employment protection for women also changed during this period. The *Special Provisions on Labor Protection for Female Employees*, which were adopted on April 18, 2012, strengthened statutory protection for female employees. Article 5 explicitly prohibited employers from reducing women’s wages, dismissing them, or terminating their employment because of pregnancy, childbirth, or breastfeeding. Earlier regulations had already prohibited reductions in female employees’ basic wages and the termination of labor contracts during these periods. The 2012 provisions therefore clarified and broadened the existing employment protections.

We construct a categorical fertility policy variable, *policy*1, to capture differences in fertility policy implementation across provinces and survey years. For each respondent and survey year, the variable equals 1 when the respondent’s province allows a second child when either spouse is an only child, 2 under the selective two-child policy, and 3 under the universal two-child policy. We also construct a binary female employment protection variable, *policy*2. For respondents who experience the relevant birth transition, policy2 equals 1 if the birth occurred in 2012 or later and 0 otherwise. Respondents who do not experience the relevant transition are coded as 0. Both variables are included as additional controls in the baseline regressions. As shown in columns (5) through (8) of Table 5, adding these policy controls produces only minor changes in the estimated coefficients, which retain the same signs and statistical significance as the baseline estimates in Table 3.

#### 4.3.4 Sensitivity to selection on unobservables

The baseline specifications include a broad set of controls, but omitted factors may still be related to both childbirth timing and annual wages. We therefore use the coefficient stability approach of Oster (2019) to examine the sensitivity of the results to this concern. The method compares the treatment coefficient and *R*^2^ across specifications with different sets of observed controls, giving a sense of how sensitive the results are to selection on unobservables. Following Oster (2019), *R*_max_ is set to the smaller of 1 and 1.3 times the *R*^2^ from the fully controlled model. Table 6 shows the coefficient bounds under *δ* = 1, where unobserved selection is assumed to be as strong as observed selection. It also gives the *δ* value required to reduce each estimate to zero.

**Table 6:** Oster sensitivity analysis for selection on unobservables.

Measure	Scheme A (first birth transition)		Scheme B (second birth transition)	
	Female	Male	Female	Male
	(1)	(2)	(3)	(4)
Identified set *δ* = 1	[−0.116, −0.107]	[0.048, 0.132]	[−0.314, −0.213]	[0.033, 0.120]
*R*_max_	0.608	0.564	0.641	0.636
*δ* required for *β* = 0	4.252	1.089	3.927	−4.936

*R*_max_ is set to the smaller of 1 and 1.3 times the *R*^2^ of the fully controlled model. The identified sets are calculated under *δ* = 1. The final row reports the value of *δ* required to reduce the estimated coefficient to zero.

The coefficient bounds keep the same signs as the baseline estimates and exclude zero under *δ* = 1. Among women, *δ* is 4.252 for the first birth transition and 3.927 for the second, so unobserved selection would need to be about four times stronger than observed selection to wipe out the estimates. For men, the first birth transition gives a *δ* of 1.089, which points to greater sensitivity to unobserved selection than the female estimates. Even then, unobserved selection would still need to be slightly stronger than observed selection to reduce it to zero. The male second birth analysis yields a negative value of *δ*, since the coefficient and *R*^2^ move in a direction inconsistent with positive proportional selection. Under the proportional selection assumption, the coefficient bounds still exclude zero, further supporting the stability of the baseline findings.

## 5 Further analyses

### 5.1 Moderation effect analysis

To assess whether the wage effects of childbirth vary with family support, we use a binary indicator coded 1 if the spouse’s income is above the sample mean or if the household reports spending on domestic services, and 0 otherwise. These two components capture household economic resources and access to paid domestic services, both of which may help families manage childcare costs and caregiving demands (Cortés and Pan, 2013). Columns (1) and (2) of Table 7 report the interaction estimates. For women, the interaction terms are positive and significant at both parity levels, indicating smaller negative wage effects in households with family support. Economic resources and access to domestic services may ease some of the constraints involved in combining paid work with childcare. The interaction terms are also positive and significant for men, with a larger fatherhood wage premium in households with family support. This may partly reflect breadwinner expectations after childbirth (Killewald, 2013; Markussen and Strøm, 2022).

**Table 7:** Moderating effects of family support and the digital environment.

Variable	Female	Male	Female	Male	Female	Male
	(1)	(2)	(3)	(4)	(5)	(6)
One child × family support	0.115^***^ (4.465)	0.057^***^ (3.034)				
Two children × family support	0.083^*^ (1.897)	0.054^**^ (2.225)				
One child × individual digital financial service use			0.038^*^ (1.871)	0.038^**^ (2.361)		
Two children × individual digital financial service use			0.058^*^ (1.664)	0.049^**^ (2.201)		
One child × regional digital finance development					0.064^***^ (2.615)	0.035^**^ (1.963)
Two children × regional digital finance development					0.082^**^ (2.362)	0.037^*^ (1.736)
Control variables	Y	Y	Y	Y	Y	Y
City FE	Y	Y	Y	Y	Y	Y
Year FE	Y	Y	Y	Y	Y	Y
Cohort FE	Y	Y	Y	Y	Y	Y
Observations	12,231	17,212	12,231	17,212	6,460	9,014
*R* ^2^	0.465	0.447	0.458	0.446	0.415	0.417

***, **, and * represent *p* < 0.01, *p* < 0.05, and *p* < 0.1, respectively. Robust *t*-statistics based on standard errors clustered at the individual level are reported in parentheses. The constituent main effects of the interaction terms are included in all specifications but are not reported. The regional digital finance development index is standardized. Columns (5) and (6) additionally include interactions of the parity indicators with centered log GDP per capita and the tertiary sector share. The reference category for childbirth status is respondents with no children.

Digital finance is measured at both the individual and regional levels. Individual use equals 1 when respondents report using digital payments such as Alipay or WeChat Pay, or purchasing financial products through apps or online platforms, and 0 otherwise. Regional digital finance is captured by the Peking University Digital Financial Inclusion Index (Guo et al., 2020), matched to the respondents’ locations and standardized before being included in the interaction specifications. To account for regional economic conditions, we also interact the birth transition indicators with centered log GDP per capita and the tertiary sector share. Columns (3)–(6) present the estimates for digital finance. For women, the interaction terms for individual digital financial service use and regional digital finance development are positive and significant for both birth transitions. Given the negative estimated wage effects for women, these coefficients indicate a smaller motherhood wage penalty among users of digital financial services and in regions with more developed digital finance. The estimates for men are also positive, although the interactions with individual digital financial service use are smaller. Easier access to financial services and lower transaction costs may help parents manage some of the financial and time pressures following childbirth. The results for family support and digital finance are consistent with H3.

### 5.2 Analysis of potential labor market channels

Childbirth may affect wages partly through changes in paid work, since fewer hours or spells out of employment can slow the accumulation of work experience and firm-specific human capital. As care responsibilities remain gendered, these adjustments are more likely to fall on women. Following Goldin (2014) and Kleven et al. (2019), we look at three potential channels: working hours, employment interruption, and career progression. Among employed respondents, working hours are based on reported weekly hours. Employment interruption is identified when respondents are currently not employed but report having worked before. Career progression is measured by whether the respondent holds a leadership or managerial position.

Table 8 reports the estimates for parity and individual labor market outcomes separately by gender. As shown in Columns (1)–(3), relative to childless women, women with one child are more likely to experience an employment interruption. At parity two, the results show shorter working hours and a higher chance of employment interruption, while the probability of holding a leadership position also declines. For men, Columns (4)–(6) indicate that those with two children work longer hours and tend to have more continuous employment than childless men. The gender contrast is clearest among respondents with two children. Unequal childcare responsibilities may leave women with less time for paid work and make it harder to remain continuously employed. Some women may also shift to more flexible jobs with shorter hours and lower wages. Breadwinner expectations may work differently for men, strengthening labor market attachment and encouraging longer working hours. Changes in working hours, employment interruption, and career progression may help account for part of the gender divergence in wages after childbirth. These associations are suggestive rather than causal, and unobserved influences related to employer responses and gender norms may also shape wage outcomes.

**Table 8:** Childbirth and potential labor market channels.

Variable	Female			Male		
	Working Hours	Employment Interruption	Career Progression	Working Hours	Employment Interruption	Career Progression
	(1)	(2)	(3)	(4)	(5)	(6)
One child	−0.246 (−0.704)	0.017^***^ (2.800)	0.003 (0.791)	0.055 (0.180)	−0.007 (−1.384)	0.005 (1.117)
Two children	−4.571^***^ (−8.751)	0.071^***^ (7.291)	−0.028^***^ (−3.319)	2.413^***^ (5.097)	−0.018^***^ (−2.648)	0.011 (1.173)
Control variables	Y	Y	Y	Y	Y	Y
City FE	Y	Y	Y	Y	Y	Y
Year FE	Y	Y	Y	Y	Y	Y
Cohort FE	Y	Y	Y	Y	Y	Y
Observations	12,231	17,677	12,231	17,212	20,463	17,212
*R* ^2^	0.173	0.109	0.074	0.171	0.086	0.121
Difference: two children − one child	−4.325^***^	0.054^***^	−0.031	2.385^***^	−0.011^**^	0.006

***, **, and * represent *p* < 0.01, *p* < 0.05, and *p* < 0.1, respectively. Robust *t*-statistics based on standard errors clustered at the individual level are reported in parentheses. The reference category for childbirth status is respondents with no children.

### 5.3 Heterogeneity analysis

#### 5.3.1 Heterogeneity by unit system

Job stability, leave arrangements, employment protection, and wage setting differ across work units and may shape the wage consequences of childbirth. Following Démurger et al. (2012), party and government organs, public institutions, and enterprises under state ownership or control are classified as within the work unit system. Table 9 reports the estimates by work unit status. The wage effects are negative for women and positive for men both within and outside the system. The motherhood wage penalty is smaller within the system at both parity levels, although the difference is significant only among women with one child. For men, the fatherhood wage premium is larger outside the system, but the group difference is significant only among those with two children. One caveat is that the wage sample covers only employed respondents, so the results for women outside the system may understate the full labor market disadvantage associated with childbirth. Greater job stability and maternity protection within the system, together with wage setting that is more closely tied to performance outside the system, may contribute to these differences.

**Table 9:** Heterogeneity by work unit type.

Panel A. Estimates by work unit type				
Variable	Female		Male	
	Within the system	Outside the system	Within the system	Outside the system
	(1)	(2)	(3)	(4)
One child	−0.092^***^ (−4.535)	−0.113^***^ (−4.488)	0.028^*^ (1.762)	0.055^**^ (2.465)
Two children	−0.257^***^ (−6.530)	−0.263^***^ (−5.141)	0.120^***^ (5.018)	0.215^***^ (6.745)
Control variables	Y	Y	Y	Y
City FE	Y	Y	Y	Y
Year FE	Y	Y	Y	Y
Cohort FE	Y	Y	Y	Y
Observations	6,994	5,184	10,337	6,585
*R* ^2^	0.452	0.451	0.418	0.464

Panel B. Tests of differences across work unit type		
Comparison	Female	Male
One child: within − outside	0.021^***^	−0.027
Two children: within − outside	0.006	−0.095^***^

***, **, and * represent *p* < 0.01, *p* < 0.05, and *p* < 0.1, respectively. Robust *t*-statistics based on standard errors clustered at the individual level are reported in parentheses. The reference category for childbirth status is respondents with no children.

#### 5.3.2 Heterogeneity by establishment status

*Bianzhi* is a distinctive employment setup in China and is common in government departments, public institutions, and organizations affiliated with the state. Compared with non-establishment positions, establishment posts are generally subject to more formal personnel rules and offer greater job security. Pay and benefits tend to be more standardized, and career progression is usually more clearly defined. Respondents who hold an establishment position in an administrative agency, public institution, or the military are classified as establishment staff, while all others are classified as non-establishment staff. Table 10 reports the results by establishment status. For women, the motherhood wage penalty is larger in absolute magnitude among non-establishment workers at both parity levels, although the gap between the groups is statistically significant only for women with two children. A similar pattern appears for the fatherhood premium, which is larger among non-establishment workers, with significant group differences at both parity levels. Greater employment stability among establishment staff may help explain the smaller wage adjustments observed after childbirth.

**Table 10:** Heterogeneity by establishment status.

Panel A. Estimates by establishment status				
Variable	Female		Male	
	Establishment staff	Non-establishment staff	Establishment staff	Non-establishment staff
	(1)	(2)	(3)	(4)
One child	−0.100^***^ (−2.89)	−0.115^***^ (−6.58)	0.030 (0.98)	0.035^***^ (2.48)
Two children	−0.147^*^ (−1.74)	−0.329^***^ (−12.33)	0.110^***^ (2.67)	0.166^***^ (8.35)
Control variables	Y	Y	Y	Y
City FE	Y	Y	Y	Y
Year FE	Y	Y	Y	Y
Cohort FE	Y	Y	Y	Y
Observations	1757	10,449	2,532	14,768
*R* ^2^	0.476	0.505	0.450	0.472

Panel B. Tests of differences across establishment status		
Comparison	Female	Male
One child: establishment − non-establishment	0.015	−0.005^**^
Two children: establishment − non-establishment	0.182^***^	−0.056^*^

***, **, and * represent *p* < 0.01, *p* < 0.05, and *p* < 0.1, respectively. Robust *t*-statistics based on standard errors clustered at the individual level are reported in parentheses. The reference category for childbirth status is respondents with no children.

### 5.4 Oaxaca–Blinder decomposition

We also use the Oaxaca–Blinder decomposition to examine the gender wage gap under Schemes A and B (Blinder, 1973; Oaxaca, 1973). For each scheme, the mean difference in log wages between men and women is separated into two parts: one part comes from differences in observed characteristics, and the other from differences in the estimated coefficients, as follows:

$${\bar{Y}}_{m}-{\bar{Y}}_{w}=({\bar{X}}_{m}-{\bar{X}}_{w}){\hat{\beta }}_{m}+{\bar{X}}_{w}({\hat{\beta }}_{m}-{\hat{\beta }}_{w})$$ (5)

In Equation 5, ${\bar{Y}}_{m}$ and ${\bar{Y}}_{w}$ represent the mean log wages of men and women, respectively, within each scheme. ${\bar{X}}_{m}$ and ${\bar{X}}_{w}$ denote the average observed characteristics of men and women, while ${\hat{\beta }}_{m}$ and ${\hat{\beta }}_{w}$ are the coefficient vectors estimated from the male and female wage equations. The first term is the explained component of the gap, which captures differences in observed characteristics evaluated using the male wage structure. The second is the unexplained component, which captures differences in estimated coefficients, including the intercept, as well as other unobserved factors. Within each scheme, we also report the contribution of the childbirth transition interaction, *treat* × *period*, within both components. For the explained part, the gender difference in the mean of the interaction term is combined with the male coefficient. The unexplained part instead uses the female mean together with the difference between the male and female coefficients.

Table 11 reports the decompositions for Schemes A and B using log annual and hourly wages. The observed mean gender wage gap is positive and statistically significant in all four specifications. The explained component, however, is consistently negative, which means that, under the male reference wage structure, observed differences in characteristics narrow rather than widen the wage gap. As a result, the unexplained component exceeds the observed gap in absolute value.

**Table 11:** Oaxaca–Blinder decomposition results.

Component	Log annual wage		Log hourly wage	
	Scheme A (first birth transition)	Scheme B (second birth transition)	Scheme A (first birth transition)	Scheme B (second birth transition)
	(1)	(2)	(3)	(4)
Difference	0.1651^***^	0.2811^***^	0.0998^***^	0.2134^***^
Explained	−0.0784^***^	−0.0565^***^	−0.0720^***^	−0.0411^***^
Contribution of treat × period	−0.0010^*^	0.0026^***^	−0.0115^***^	0.0024^***^
Unexplained	0.2434^***^	0.3376^***^	0.1718^***^	0.2545^***^
Contribution of treat × period	0.1208^***^	0.0430^***^	0.1122^***^	0.0338^***^

***, **, and * represent *p* < 0.01, *p* < 0.05, and *p* < 0.1, respectively. The decomposition uses the male wage structure as the reference structure.

In Scheme A, the *treat* × *period* term makes a negative contribution to the explained component, while its contribution to the unexplained component is positive and statistically significant. For the second birth comparison, both components are positive and statistically significant. Under Scheme A, the childbirth transition term makes up a large part of the unexplained component, about half of it for annual wages and close to two thirds for hourly wages. Its role is smaller under Scheme B, both in absolute size and as a share of the unexplained component, even though the unexplained component itself is larger. The unexplained component may capture aspects of wage determination that observed characteristics do not reflect, such as potential gender discrimination, labor market segmentation, differences in wage setting, and gender norms around paid work and childrearing (Becker, 1957; Phelps, 1972; Blau and Kahn, 2017; Kleven, 2022). Coefficient differences outside the childbirth transition term therefore carry more weight in the second birth comparison and may be related to cumulative labor market disadvantages associated with larger family size.

### 5.5 Analysis of childbirth and the gender earnings gap within couples

Gendered household norms may shape how couples divide paid work and childcare. This section further examines the association between childbirth transitions and the earnings gap between spouses. Childbirth may shift household labor from paid employment into unpaid childcare and domestic work. Since wages are observed only for those who are employed, they miss employment exits and other changes in labor force participation that may accompany this shift. We therefore rely on earnings rather than wages in this analysis. Earnings are coded as zero for individuals who are not employed. The within couple earnings gaps are calculated as the husband’s earnings minus the wife’s earnings for both annual and hourly measures. Positive values mean the husband earns more, while negative values indicate the reverse. With non-employed spouses coded as zero, these measures reflect both employment participation and earnings differences within couples.

Table 12 shows that, under Scheme A, the first birth transition is associated with an increase of CNY 5,213.404 in the annual earnings gap and CNY 2.199 in the hourly earnings gap between spouses. For Scheme B, the corresponding increases are CNY 10,311.771 and CNY 4.008, respectively, with all four estimates significant at the 1% level. The larger estimates point to a wider earnings gap between spouses in the second birth comparison. This widening may reflect a gendered reallocation of household labor between paid and unpaid work. If women reduce paid work while taking on more childcare and domestic responsibilities, the resulting decline in market earnings need not mean that their total productive contribution has fallen, because unpaid household work is not captured in market earnings.

**Table 12:** Childbirth and the gender earnings gap within couples.

Variable	Scheme A		Scheme B	
	Annual earnings gap	Hourly earnings gap	Annual earnings gap	Hourly earnings gap
	(1)	(2)	(3)	(4)
Treat × Period	5213.404^***^ (4.358)	2.199^***^ (3.307)	10311.771^***^ (6.588)	4.008^***^ (4.909)
Control variables	Y	Y	Y	Y
City FE	Y	Y	Y	Y
Year FE	Y	Y	Y	Y
Cohort FE	Y	Y	Y	Y
Observations	11,227	11,227	10,170	10,170
*R* ^2^	0.059	0.047	0.088	0.065

***, **, and * represent *p* < 0.01, *p* < 0.05, and *p* < 0.1, respectively. Robust *t*-statistics based on standard errors clustered at the individual level are reported in parentheses. The earnings gap is defined as the husband’s earnings minus the wife’s earnings. Annual and hourly earnings are coded as zero for non-employed spouses.

## 6 Conclusion

Gender wage inequality remains an important labor market concern, and childbirth may shape the wage trajectories of women and men differently. China’s gradual relaxation of fertility policy offers the institutional setting for examining whether these wage differences vary by birth order. This study examines the impact of the first and second birth transitions on the gender wage gap across different groups, using data from the 2013, 2015, 2017, and 2019 waves of the CHFS. The main findings are as follows.

First, both birth transitions are associated with lower wages for women and higher wages for men, consistent with a motherhood wage penalty and a fatherhood wage premium. The estimated wage changes are larger for the second birth transition than for the first. Second, family support and digital finance moderate the estimated wage effects of childbirth. Changes in working hours, employment interruption, and career progression point to possible channels behind the gender gap in wage responses. Third, the estimated effects vary by work unit status and establishment status, with generally larger absolute estimates among workers outside the work unit system and those without establishment status. Fourth, the Oaxaca–Blinder decomposition shows that observed characteristics narrow the mean gender wage gap, whereas the unexplained component widens it. Within couples, both birth transitions are also tied to a wider earnings gap between husbands and wives, with larger point estimates in the second birth comparison.

The results carry several implications for policy. The larger wage reductions for women, particularly after the second birth, together with changes in working hours and employment continuity, point to the importance of supporting women’s continued employment after childbirth. The smaller wage penalties observed in settings with stronger employment protection are consistent with a buffering role for such protection. Affordable childcare and greater paternal involvement may also ease work and family constraints, as suggested by prior studies. The moderating patterns suggest that the wage consequences of childbirth also vary with household resources, individual use of digital financial services, and regional digital finance development.

The analysis also has limitations. The four CHFS waves only offer a limited window around childbirth, and fertility timing may still be related to unobserved individual or household traits. Future studies using longer panel data could follow these wage differences over time and examine whether they persist or accumulate as family size and children’s ages change.

## Data Availability

The data analyzed in this study is subject to the following licenses/restrictions: the data analyzed in this study were obtained from the China Household Finance Survey. Restrictions apply to the availability of these data, which may be accessed upon application to the China Household Finance Survey and Research Center. Requests to access these datasets should be directed to China Household Finance Survey and Research Center, Southwestern University of Finance and Economics: https://chfser.swufe.edu.cn/data/.

## Footnotes

1 The Fourth Survey on the Social Status of Chinese Women shows that while 91.1% of couples make childbearing decisions together, mothers primarily shoulder the responsibilities of daily care, homework assistance, and transportation for children aged 0–17, accounting for 76.1%, 67.5%, and 63.6%, respectively.

2 Article 6 of the *Marriage Law of the People’s Republic of China*, applicable during the study period, set the minimum marriage age at 22 for men and 20 for women. According to the provisions of the “Interim Measures of the State Council on the Placement of Old, Weak, Sick, and Disabled Cadres” and the “Interim Measures of the State Council on the Retirement and Resettlement of Workers,” the statutory retirement ages applicable during the survey period were 60 years for men, 55 years for female cadres, and 50 years for female employees.

3 According to the *Labor Law of the People’s Republic of China* and the *Provisions on the Composition of Total Wages*, wages refer to labor remuneration paid by employers and generally include time-rate and piece-rate wages, bonuses, allowances and subsidies, overtime pay, and wages paid under special circumstances. Annual wage is therefore constructed as the sum of three items reported in the CHFS: after tax cash wages, after tax bonuses, and after tax subsidies or in kind remuneration. This measure approximates the main components of wage remuneration under the Chinese definition.

4 Cohort fixed effects are defined by province and five-year birth cohort, with respondents assigned to five-year cohorts according to their year of birth.

5 Other household income equals total household income minus the respondent’s annual income and is transformed using ln(other household income + 1).

6 For example, Shandong, Sichuan, and 27 other provinces, municipalities, and districts completed the transition from the “one-child policy” to the “two-child fertility policy for couples where either is from a single-child family” before 2000. Henan Province began implementing the “two-child fertility policy for couples where either is from a single-child family” in 2011.
